# Community Pharmacy‐Based Injectable Opioid Agonist Treatment: Findings From a Canadian Pilot Program

**DOI:** 10.1111/dar.70062

**Published:** 2025-11-04

**Authors:** Tamara Mihic, Maria Eugenia Socias, Karen McCrae, Cheyenne Johnson, Seonaid Nolan, Cameron Grant, Christy Sutherland, Nadia Fairbairn

**Affiliations:** ^1^ British Columbia Centre on Substance Use Vancouver British Columbia Canada; ^2^ Faculty of Pharmaceutical Sciences University of British Columbia Vancouver British Columbia Canada; ^3^ Department of Medicine University of British Columbia Vancouver British Columbia Canada

**Keywords:** community pharmacy services, opiate substitution treatment, opioid‐related disorders

## Abstract

**Introduction:**

Access to evidence‐based treatment for opioid use disorder remains limited, particularly for individuals who have not responded to oral opioid agonist treatment (OAT). A community pharmacy‐based model of injectable OAT (iOAT) was piloted in Vancouver, Canada from March 2017 to December 2018. This brief report describes the program structure, participant sociodemographics, reported outcomes, and strengths and areas for improvement of the program.

**Methods:**

A retrospective review of cross‐sectional, interviewer‐led questionnaire data from participants who accessed iOAT at the pharmacy site (*n* = 176) and provided informed consent was conducted. Outcomes include participant‐reported changes in symptomatology, function and satisfaction, analysed through descriptive statistics. Open‐ended responses were analysed using content analysis to identify strengths and areas for improvement of the program.

**Results:**

Fifty‐one participants (29%) completed the questionnaire, and most had multiple previous overdoses and trials of oral OAT. The most commonly reported outcomes were reduction in illicit opioid use (76%), opioid cravings (45%) and illicit substance use (45%). Participants identified key strengths of the program as positive experiences with staff and efficiency of the pharmacy model including flexible dosing time and the ability to pick up other medications at the same time. Suggested improvements focused on medication options (e.g., access to diacetylmorphine, alternate routes of administration), expanded hours and flexibility, additional support services, and increased capacity and space.

**Discussion and **Conclusions**:**

Community pharmacy‐based iOAT represents a novel strategy to expand access to evidence‐based opioid use disorder treatment among individuals who inject opioids and have not responded to or do not prefer oral OAT.

**Summary**

- Community pharmacy‐based models offer a novel strategy to expand access to injectable opioid agonist treatment.
- Participants had multiple previous overdoses and trials of oral opioid agonist treatment.
- Participants reported reduction in illicit opioid use, cravings and illicit substance use.
- Participants reported positive experiences with staff and efficiency of the community pharmacy model.
- Areas for improvement included program rules, addition of diacetylmorphine and expanding physical space.

## 1 Introduction

The on‐going opioid overdose crisis, which has contributed to over 600,000 deaths in the USA and Canada since 1999, highlights the critical need to expand evidence‐based treatments for opioid use disorder [1, 2]. Oral opioid agonist treatment (OAT), such as methadone and buprenorphine/naloxone, has been shown to reduce illicit drug use, overdose and mortality. However, treatment retention and relapse remain ongoing challenges [3, 4, 5]. Injectable OAT (iOAT), supervised injection of diacetylmorphine or hydromorphone multiple times a day, has demonstrated further reduction in illicit opioid use and treatment discontinuation compared to oral OAT [6, 7, 8, 9]. Despite the availability of iOAT programs through clinic settings since 2005 [2], more than 395 Canadians were on waitlists as of 2018, with most programs concentrated in urban centres [10]. To expand capacity and access, provision through a community pharmacy was explored. This approach could increase enrolment, extend reach to underserved areas, and offer patients more flexible dosing and the convenience of receiving all medications in one location.

A community pharmacy‐based model was piloted from March 2017 to December 2018 at a single community pharmacy in Vancouver, British Columbia (BC). Participants were identified through a single outpatient clinic providing a variety of primary care and substance use services, titrated to a therapeutic dose of injectable hydromorphone by a physician within a clinic (nurse‐supervised injection), then transitioned to a single community pharmacy site for supervised dose administration up to three times daily. Only hydromorphone was offered due to regulatory constraints limiting access to diacetylmorphine [11]. The program was integrated into usual pharmacy practice. Participants could present at any time during pharmacy hours (8:30 AM–5:00 PM) and self‐inject under the supervision of a pharmacist or pharmacy‐employed nurse, followed by at least 15 min of observation. Oral OAT, including methadone or slow‐release oral morphine, could also be dispensed by the pharmacist, as well as medications for other chronic medical conditions. Patients were still followed at the clinic for ongoing medical follow‐up, OAT prescribing, and additional support services. Additional information about overall iOAT services in BC, including the community pharmacy‐based model has previously been published [12]. The objectives of this brief report are to describe participant‐reported sociodemographics of individuals accessing iOAT through the community pharmacy; participant‐reported outcomes of community pharmacy‐based iOAT treatment; and participant‐reported strengths and areas for improvement of the community pharmacy‐based iOAT program.

## 2 Methods

As part of a larger ongoing mixed‐methods prospective cohort study of patients accessing iOAT programs in BC, interviewer‐administered baseline and 12‐month questionnaires of participants accessing iOAT at any site in BC were completed in person starting April 2018 [13]. Participants provided informed consent and were able to refuse any question without justification and could elect to terminate the interview at any stage. Care was taken to ensure that clients who did not wish to participate in this evaluation were not precluded from accessing treatment in any way. The questionnaire included a variety of question types including multiple‐choice, Likert scale and options for open‐ended answers. The questionnaire took approximately 1–1.5 h to complete and participants received $40 for their time. Baseline urine drug test results, available for some participants through the clinic as part of provincial iOAT program requirements at the time [14], were used to objectively characterise substance exposure (including fentanyl and other substances) in the context of the toxic and unpredictable unregulated drug supply.

For this sub‐analysis, consenting individuals 19 years of age or older, who accessed iOAT at the single community pharmacy site (available until December 2018) and responded to at least one question from the questionnaire were included in the data analysis.

Participant outcomes were categorised using Proctor et al.'s implementation science framework [15], which includes “client outcomes” of symptomatology, function and satisfaction. Participant‐reported outcomes included:

*Symptomatology*

1. Changes in substance use (e.g., reduced/stopped use of opioids or other substances).
2. Health outcomes (e.g., physical and mental health, overdose reduction).
3. Adverse effects of iOAT.

*Function*

4 Social functioning outcomes (e.g., housing, income, criminal justice involvement).

*Satisfaction*

5 Satisfaction with services (e.g., whether participants would return to the program, would recommend it to others, and their overall satisfaction with the program).

Results were analysed using descriptive statistics reporting the proportion of participants responding “yes” or “no” to each question. Non‐response to a question and refusal to respond were grouped together as “unknown”.

Two questions related to strengths and areas for improvement of the pharmacy‐based iOAT program with open‐ended responses were reviewed using a content analysis. Each individual response was recorded under the following categories: program staff; medication (including type of medication, dose, route of administration, take‐home dosing/deliveries); physical space (e.g., location, size of the injection space, privacy); program accessibility, rules, and workflow; program capacity; and additional supports (e.g., connection to other programs and services). Multiple responses may have been identified from each participant and may have been included under the same category (e.g., mention of increase to TID dosing and offering diacetylmorphine both included under “medication”).

## 3 Results

### 3.1 Participant Sociodemographic Factors and History of Drug Use

Of 176 participants who accessed the pharmacy‐based iOAT program, 51 completed the questionnaire (29%). The median age was 53 years. Most participants identified as a man (*n* = 44, 86%) and approximately half (*n* = 27, 53%) identified as White. Almost all participants lived in an urban setting (*n* = 46, 90%) and over half (*n* = 33, 65%) reported being unstably housed. Social assistance (*n* = 42, 84%) and informal, illegal or prohibited sources (*n* = 33, 65%) were the most commonly reported sources of income. More than half of participants (*n* = 29, 57%) had a prior history of being in jail, prison or on parole. Common comorbidities included hepatitis C (*n* = 31, 61%) and mental health conditions (*n* = 22, 43%).

Most participants (*n* = 37, 73%) reported a history of overdose, with a median of five prior overdoses. Prior to iOAT initiation, 33 participants (85%) reported previous trials of oral OAT. Of 44 participants with baseline urine drug test results, 31 (70%) were positive for fentanyl, and stimulants were the most common additional non‐opioid substances identified (amphetamine 82%, cocaine 27%). Further sociodemographic factors and substance use history are described in Table 1.

**TABLE 1:** Baseline characteristics of participants in the iOAT pharmacy program in Vancouver, Canada between March 2017 and December 2018.

	Total (*n* = 51) *n* (%)
Median age in years (IQR)	53 (43–58)
Male sex	45 (88.2)
Gender	
Man	44 (86.3)
Woman	7 (13.7)
Other	0 (0.0)
Race	
White	27 (52.9)
Black, Indigenous or other person of colour	19 (37.3)
Unknown	5 (9.8)
Sexual orientation	
Heterosexual	44 (86.3)
Homosexual	1 (2.0)
Other	5 (9.8)
Unknown	1 (2.0)
Location	
Vancouver	46 (90.2)
Lower mainland	2 (3.9)
Unknown	3 (5.9)
Housing type	
House or apartment	14 (27.5)
Single room occupancy hotel	16 (31.4)
Shelter	9 (18.0)
No fixed address	4 (7.8)
Other	4 (7.8)
Unknown	4 (3.9)
Sources of income in last 6 months*	
Employed	8 (15.7)
Retired	1 (1.2)
Social assistance	42 (82.3)
Sex work	1 (0.5)
Illegal activity	18 (35.3)
Other (e.g., recycling, pan handling, from friends, etc.)	15 (29.3)
Ever in jail	
Yes	29 (56.9)
No	7 (13.7)
Unknown	15 (29.4)
Comorbidities*	
Mental health conditions	22 (43.1)
Hepatitis C	31 (60.8)
HIV	9 (17.7)
COPD	9 (17.7)
Heart disease	6 (11.8)
Diabetes	3 (5.9)
Other	10 (19.6)
None	5 (9.8)
Prior opioid overdose	
Yes	37 (72.5)
No	8 (15.7)
Unknown	6 (11.8)
Median number of prior overdoses (IQR)	5.0 (3.0–10.5)
Hospitalised in the last 6 months	
Yes	16 (31.4)
No	16 (31.4)
Unknown	19 (37.3)
Opioid used most often prior to iOAT	
Heroin	18 (78.3)
Fentanyl	3 (13.0)
Other	2 (8.7)
Unknown	27 (54.0)
Other substance use	
Stimulants	33 (86.7)
Cannabis	1 (2.6)
Alcohol	0 (0.0)
Other	0 (0.0)
Unknown	12 (24.0)
Location of drug use prior to iOAT	
Indoor public space	2 (3.9)
Overdose prevention site/supervised consumption site	8 (15.7)
Outdoor public space	3 (5.9)
Indoor private space	21 (41.2)
Other	2 (3.9)
Unknown	15 (29.4)
Previous OAT*	
Methadone	27 (52.9)
Buprenorphine/naloxone	19 (37.3)
Sustained‐release oral morphine	17 (33.3)
No prior OAT	6 (11.8)
Unknown	12 (23.5)

Abbreviations: COPD, chronic obstructive pulmonary disease; HIV, human immunodeficiency virus; iOAT, injectable opioid agonist treatment; IQR, interquartile range; OAT, opioid agonist treatment; SRO, single room occupancy.

* Respondents may have selected more than one option.

### 3.2 Participant‐Reported Outcomes

Most participants received 2 doses of iOAT at the pharmacy each day (*n* = 42, 82%), and 42 participants (82%) received concurrent OAT (*n* = 30 slow‐release oral morphine and *n* = 12 methadone). Participant‐reported outcomes associated with participation in the community pharmacy‐based iOAT program are summarised in Table 2.

**TABLE 2:** Participant‐reported outcomes from the iOAT pharmacy model in Vancouver, Canada between March 2017 and December 2018.

Outcome	Yes *n* (%)	No *n* (%)	Unknown *n* (%)
Symptomatology			
Reduced cravings	23 (45)	3 (6)	25 (49)
Stopped illicit use of opioids	10 (20)	32 (63)	9 (18)
Stopped non‐medical use of prescription opioids	6 (12)	8 (16)	37 (73)
Reduced illicit use of opioids	39 (76)	4 (8)	8 (16)
Stopped illicit use of other substances	6 (12)	19 (37)	26 (51)
Reduced illicit use of other substances	23 (45)	16 (31)	12 (24)
Reduced or stopped having opioid overdoses	19 (37)	21 (41)	11 (22)
Improved pain management	16 (31)	2 (4)	33 (65)
Improved physical health	18 (35)	2 (4)	31 (61)
Improved mental health	15 (29)	4 (8)	32 (63)
Improved overall health	13 (25)	9 (18)	29 (57)
Function			
Improved housing	3 (6)	34 (67)	9 (18)
Improved food security	23 (45)	19 (37)	9 (18)
Improved income via start of income assistance	8 (16)	34 (67)	9 (18)
Improved income via reduced spending on drugs	33 (65)	9 (18)	9 (18)
Improved income via paid work	5 (10)	35 (67)	11 (22)
Reduced or stopped illegal income generating activities	21 (41)	18 (35)	12 (24)
Less threat of physical assault/violence	10 (20)	6 (12)	35 (67)
Stopped being arrested or going to jail	16 (31)	21 (41)	14 (27)
Reconnected with family or friends	13 (25)	29 (57)	9 (18)
Went to school or participated in educational activities	4 (8)	36 (71)	11 (22)
Felt less stigma on treatment	9 (18)	30 (59)	12 (24)
Provided stability in life	17 (33)	4 (8)	30 (59)
Satisfaction			
Received the kind of service they wanted	12 (24)	4 (8)	35 (69)
Would they come back to the program	10 (20)	3 (6)	38 (74)
Would they recommend the program to a friend	13 (25)	2 (4)	36 (71)
In general, are they satisfied with the service	11 (22)	3 (6)	37 (73)

#### 3.2.1 Symptomatology

Most commonly participants reported a reduction in illicit opioid use (39, 76%), opioid cravings (23, 45%) and use of other illicit substances (23, 45%). Many participants also reported a reduction in opioid overdoses (19, 37%), improved physical (18, 35%) and mental health (15, 29%), and improved pain management (16, 31%). A total of 8 individuals reported a drug toxicity (i.e., overdose) event while in receipt of iOAT; all of these events occurred outside of the study site and were not secondary to iOAT administration. Most commonly reported adverse effects of hydromorphone iOAT included feeling “pins and needles” around the injection site (7, 14%) and symptoms suggestive of a histamine reaction (e.g., pruritus, flushing, hypotension) (7, 14%).

#### 3.2.2 Function

Participants endorsed reduced drug spending (33, 65%), illegal income‐generating activities (21, 41%) and arrests and incarceration (16, 31%); improved food security (23, 45%); and overall stability in their life (17, 33%).

#### 3.2.3 Satisfaction

Of the 14 participants who provided a response, 11 (22%) reported being satisfied with the program and 13 (25%) would recommend the program to a friend.

### 3.3 Program Strengths and Areas for Improvement

**FIGURE 1:**
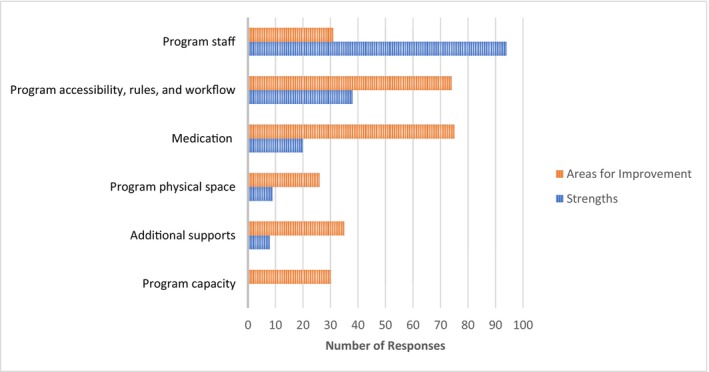
Participant reported strengths and areas for improvement of the injectable opioid agonist treatment pharmacy model in Vancouver, BC between March 2017 and December 2018.

The most commonly reported strengths of the program were related to program staff and program accessibility, rules, and workflow (Figure 1). Participants reported that staff were friendly, supportive, professional, created a positive atmosphere and provided individualised support. They also reported the program was accessible and efficient with flexible dosing (option to receive 2 doses rather than 3), program workflow and the ability to receive doses quickly, as well as the ability to pick up other medications from the pharmacy at the same time. Participants also reported medication‐related strengths particularly with quickly achieving an effective dose for symptom management.

The most common areas for improvement were related to changes to medication including offering diacetylmorphine, increasing the number of doses, offering alternate routes of administration (e.g., inhaled), and allowing for take‐home doses/carries; followed by changes to program accessibility, rules, and workflow (e.g., expanded hours for the program, options for when the pharmacy is closed on long weekends, staff consistently enforcing program rules, and more security presence); requests for additional supports (e.g., housing and employment); suggestions for staff including further education on addiction and stigma, and inclusion of peer support workers; expanding program capacity and the number of locations of the program (some participants had to travel further); and finally expanding the physical space so that it is not so crowded when waiting and more patients can inject at one time.

## 4 Discussion

This brief report is the first to describe a community‐pharmacy‐based iOAT model. Most participants identified as male between 20 and 59 years of age, which reflects the group most represented in overdose deaths in Canada [16]. Participants represented a socio‐structurally vulnerable population who may not have access to traditional models of care delivery. Substance‐use risk profile included high rates of exposure to fentanyl, polysubstance use, use of substances in private residence and multiple previous overdoses, highlighting the potential of community pharmacy‐based iOAT programs to reach populations most at risk of opioid overdose.

Participants reported improvement in symptomatology (including reduced illicit opioid use and improved physical and mental health) and functioning (including reduction in spending on drugs and illegal income generation), consistent with findings from studies of iOAT programs within clinics [17]. Rates of adverse effects were also in keeping with previous studies of hydromorphone iOAT [18].

Most respondents reported satisfaction with the program. Unique strengths included the ability to pick up other medications concurrently and increased dosing flexibility. Given the need for multiple daily witnessed doses, offering iOAT through a community pharmacy may enhance accessibility for patients by providing a more convenient and flexible alternative. This model could be particularly useful in rural or underserved areas, where pharmacies are often one of the few available healthcare services.

However, several areas for improvement were identified including the availability of diacetylmorphine, alternative routes of administration, longer hours, expanded space, education around addiction/stigma for staff, peer involvement, and additional support services. While many of these are modifiable, the pharmacy model offered fewer integrated social supports and participants remained reliant on the clinic for certain aspects of care, reflecting a trade‐off between accessibility and comprehensive care.

Barriers for expansion of the pharmacy‐based iOAT program pilot included lack of access to diacetylmorphine, requirement for multiple doses every day, variable injection time, need for pre‐ and post‐ assessment by pharmacy staff, and a private room for injection. Costs for space, equipment, and staffing were borne by the pharmacy and the standard medication dispensing fees made this financially challenging. Therefore, the pilot program was not extended after 2018 due to these logistic and financial constraints. Further exploration of ways in which to reduce these barriers may expand access to this novel model of care, particularly amid ongoing high overdose mortality and a strained healthcare system.

This descriptive program evaluation has several limitations. Data was collected through a voluntary cross‐sectional interviewer‐led questionnaire, which may introduce self‐selection, recall, response/non‐response and social desirability bias. Some participants did not answer all questions limiting inference about the population as a whole. There was also a lack of a comparator group and findings may not be generalisable to non‐urban settings. Future studies should compare pharmacy‐based iOAT to other models and expand to other populations.

## 5 Conclusion

This evaluation of a community pharmacy‐based iOAT program highlights its potential to reduce illicit opioid use, improve health and stability, and offer a more accessible alternative to clinic‐based models. While participant outcomes were generally positive, opportunities for program improvement were identified and further research is required to assess how this model compares to embedded iOAT clinics.

## Author Contributions

Each author certifies that their contribution to this work meets the standards of the international committee of medical journal editors.

## Conflicts of Interest

The authors declare no conflicts of interest.

## Data Availability

Research data are not shared.

## References

[1] K. Humphreys , C. L. Shover , C. M. Andrews , et al., “Responding to the Opioid Crisis in North America and Beyond: Recommendations of the Stanford‐Lancet Commission,” Lancet 399, no. 10324 (2022): 555–604, 10.1016/S0140-6736(21)02252-2. 35122753 PMC9261968

[2] E. Oviedo‐Joekes , S. Brissette , D. C. Marsh , et al., “Diacetylmorphine Versus Methadone for the Treatment of Opioid Addiction,” New England Journal of Medicine 361, no. 8 (2009): 777–786, 10.1056/NEJMoa0810635. 19692689 PMC5127701

[3] J. Klimas , M. A. Hamilton , L. Gorfinkel , A. Adam , W. Cullen , and E. Wood , “Retention in Opioid Agonist Treatment: A Rapid Review and Meta‐Analysis Comparing Observational Studies and Randomized Controlled Trials,” Systematic Reviews 10, no. 1 (2021): 216, 10.1186/s13643-021-01764-9. 34362464 PMC8348786

[4] A. M. O'Connor , G. Cousins , L. Durand , J. Barry , and F. Boland , “Retention of Patients in Opioid Substitution Treatment: A Systematic Review,” PLoS One 15, no. 5 (2020): e0232086, 10.1371/journal.pone.0232086. 32407321 PMC7224511

[5] M. Piske , H. Zhou , J. E. Min , et al., “The Cascade of Care for Opioid Use Disorder: A Retrospective Study in British Columbia, Canada,” Addiction 115, no. 8 (2020): 1482–1493, 10.1111/add.14947. 31899565

[6] M. Ferri , M. Davoli , and C. A. Perucci , “Heroin Maintenance for Chronic Heroin‐Dependent Individuals,” Cochrane Database of Systematic Reviews 2011, no. 12 (2011): CD003410, 10.1002/14651858.CD003410.pub4. 22161378 PMC7017638

[7] J. Strang , T. Groshkova , A. Uchtenhagen , et al., “Heroin on Trial: Systematic Review and Meta‐Analysis of Randomised Trials of Diamorphine‐Prescribing as Treatment for Refractory Heroin Addiction,” British Journal of Psychiatry 207, no. 1 (2015): 5–14, 10.1192/bjp.bp.114.149195. 26135571

[8] E. Oviedo‐Joekes , D. Guh , S. Brissette , et al., “Hydromorphone Compared With Diacetylmorphine for Long‐Term Opioid Dependence: A Randomized Clinical Trial,” JAMA Psychiatry 73, no. 5 (2016): 447–455, 10.1001/jamapsychiatry.2016.0109. 27049826

[9] E. Jozaghi , ““SALOME Gave My Dignity Back”: The Role of Randomized Heroin Trials in Transforming Lives in the Downtown Eastside of Vancouver, Canada,” International Journal of Qualitative Studies on Health and Well‐Being 9, no. 1 (2014): 23698, 10.3402/qhw.v9.23698. 24646474 PMC3955773

[10] E. Eydt , S. Glegg , C. Sutherland , et al., “Service Delivery Models for Injectable Opioid Agonist Treatment in Canada: 2 Sequential Environmental Scans,” CMAJ Open 9, no. 1 (2021): E115–E124, 10.9778/cmajo.20200021. PMC8034381 33622764

[11] Canada H , “Reducing Regulatory Barriers to Accessing Treatment, and New Funding for Innovative Projects,” 2018, https://www.canada.ca/en/health‐canada/news/2018/03/reducing‐regulatory‐barriers‐to‐accessing‐treatment‐and‐new‐funding‐for‐innovative‐projects.html.

[12] S. Mayer , N. Fairbairn , A. Fowler , J. Boyd , T. Kerr , and R. McNeil , “Barriers and Facilitators to Injectable Opioid Agonist Treatment Engagement Within a Structural Vulnerability Context: A Qualitative Study of Patient Experiences in Vancouver, Canada,” Harm Reduction Journal 22 (2025): 116, 10.1186/s12954-025-01262-4. 40615907 PMC12228173

[13] BC Centre on Substance Use , “iOAT & TiOAT Study. BCCSU,” 2024, https://www.bccsu.ca/ioat‐tioat‐study/.

[14] British Columbia Centre on Substance Use , “Guidance for Injectable Opioid Agonist Treatment for Opioid Use Disorder,” 2018, https://www.bccsu.ca/wp‐content/uploads/2021/07/BC_iOAT_Guideline.pdf.

[15] E. Proctor , H. Silmere , R. Raghavan , et al., “Outcomes for Implementation Research: Conceptual Distinctions, Measurement Challenges, and Research Agenda,” Administration and Policy in Mental Health 38, no. 2 (2011): 65–76, 10.1007/s10488-010-0319-7. 20957426 PMC3068522

[16] Government of Canada , “Opioid‐and Stimulant‐related Harms in Canada: Key findings—Canada.ca,” 2019, https://health‐infobase.canada.ca/substance‐related‐harms/opioids‐stimulants/.

[17] J. Bell , V. Belackova , and N. Lintzeris , “Supervised Injectable Opioid Treatment for the Management of Opioid Dependence,” Drugs 78, no. 13 (2018): 1339–1352, 10.1007/s40265-018-0962-y. 30132259

[18] E. Oviedo‐Joekes , H. Palis , D. Guh , et al., “Adverse Events During Treatment Induction With Injectable Diacetylmorphine and Hydromorphone for Opioid Use Disorder,” Journal of Addiction Medicine 13, no. 5 (2019): 354–361, 10.1097/ADM.0000000000000505. 30747750 PMC6791495

